# Low-Cost Plant-Based Metal and Metal Oxide Nanoparticle Synthesis and Their Use in Optical and Electrochemical (Bio)Sensors

**DOI:** 10.3390/bios13121031

**Published:** 2023-12-15

**Authors:** Iulia Corina Ciobotaru, Daniela Oprea, Constantin Claudiu Ciobotaru, Teodor Adrian Enache

**Affiliations:** 1 National Institute of Materials Physics, 405A Atomistilor, 077125 Magurele, Romania; corina.ciobotaru@infim.ro (I.C.C.); daniela.oprea@infim.ro (D.O.); claudiu.ciobotaru@infim.ro (C.C.C.); 2 Faculty of Physics, University of Bucharest, 405 Atomistilor, 077125 Magurele, Romania

**Keywords:** green synthesis, metal nanoparticles, plant extracts, optical (bio)sensors, electrochemical (bio)sensors

## Abstract

Technological progress has led to the development of analytical tools that promise a huge socio-economic impact on our daily lives and an improved quality of life for all. The use of plant extract synthesized nanoparticles in the development and fabrication of optical or electrochemical (bio)sensors presents major advantages. Besides their low-cost fabrication and scalability, these nanoparticles may have a dual role, serving as a transducer component and as a recognition element, the latter requiring their functionalization with specific components. Different approaches, such as surface modification techniques to facilitate precise biomolecule attachment, thereby augmenting recognition capabilities, or fine tuning functional groups on nanoparticle surfaces are preferred for ensuring stable biomolecule conjugation while preserving bioactivity. Size optimization, maximizing surface area, and tailored nanoparticle shapes increase the potential for robust interactions and enhance the transduction. This article specifically aims to illustrate the adaptability and effectiveness of these biosensing platforms in identifying precise biological targets along with their far-reaching implications across various domains, spanning healthcare diagnostics, environmental monitoring, and diverse bioanalytical fields. By exploring these applications, the article highlights the significance of prioritizing the use of natural resources for nanoparticle synthesis. This emphasis aligns with the worldwide goal of envisioning sustainable and customized biosensing solutions, emphasizing heightened sensitivity and selectivity.

## 1. Introduction

In recent years, the development of eco-friendly and cost-effective methodologies for the fabrication of nanoparticles (NPs) has gained significant attention in the field of nanotechnology, biotechnology and other connected domains. These nanoscale materials, compared to their bulk corresponding item, have immense potential in various applications, such as medical and biological, including biosensing for medical and environmental monitoring [1,2,3]. As per the International Organization for Standardization (ISO) and American Society for Testing and Materials (ASTM) standards [4,5], nanoparticles are particles with sizes ranging from 1 to 100 nm, and due to their small size, NPs display enhanced reactivity, strength, sensitivity and stability compared to their larger counterparts [6].

The synthesis of tailored-size and -shape NPs can be achieved through physical, chemical or biological methods. Whereas the physical and chemical routes have drawbacks such as high energy consumption, low yield, high cost, and environmental damage due to harsh reducing agents, the biological methods offer a greener alternative and involve the use of either microorganisms or plants, the first one requiring large cultures and carrying the risk of environmental contamination. Thus, focus on the large-scale fabrication of NPs for the development of innovative products, equally beneficial to human health and the environment, shifted toward green synthesis (GS) using plants, mainly for its cost-effectiveness and eco-friendliness advantages [7,8]. Indeed, there are also high-cost and environmentally harmful extraction methods, but infusion or acidified methanolic extraction, which come with large yield at a low cost, are preferred. These advantages extend beyond environmental sustainability, also being simple, scalable, and readily implemented. Also, plant parts that can be used for extraction are available for free all over places. Indeed, the synthesis of nanoparticles with one substance is much more controlled and precise than the synthesis of nanoparticles with different substances in an extract, but this comes with a high cost and is not something scalable. Low-cost GS methods employ biocompatible materials to produce NPs with desirable properties, and several metallic nanoparticles (MNPs) are specifically used because of their unique optical properties in conjunction with the possibility of being synthesized through biological methods [9]. The green synthesis of MNPs has emerged as a promising approach, utilizing the metabolites of plants, to reduce metallic ions into neutral atoms without involving toxic chemicals. Furthermore, compared to chemically synthesized MNPs, GS offers enhanced biocompatibility, making it well-suited for biomedical applications. Various types of MNPs, including gold (Au), silver (Ag), zinc (Zn) or zinc oxide (ZnO), nickel (Ni) or nickel oxide (NiO) have been synthesized using different plant extracts as reducing and, at the same time, as capping agents, rendering the resulting MNPs with high stability and biological affinities. However, there is still a lack of comprehensive understanding regarding the mechanism of action for different natural reducing agents. Even though MNPs hold tremendous application potential across various sectors, they are also admitted as a major challenge associated with toxicity, and consequently, a need for translational research to bridge the gap between laboratory synthesis and practical applications [10,11].

Utilizing GS methods in conjunction with noble and non-noble MNPs could be one way to address the issue of using toxic chemicals in traditional synthesis methods. Often, this approach fosters the creation of cutting-edge chemical and biological sensors designed to recognize both chemical and biological substances. For these enhanced sensors, environmentally friendly GS methods can be utilized to produce nanomaterials, including zero-valent noble MNPs, metal oxide nanoparticles (MONPs), and carbon-based nanomaterials. These materials find utility in crafting eco-friendly electrochemical sensors and biosensors, enabling the measurement of various molecules in commercial products and biological samples, and proving the promising potential of green-synthesized MNPs [12,13,14].

An example where the use of MNPs led to an improve in performances is represented by the electrochemical biosensors. These analytical devices combine the selectivity of biological recognition elements, such as enzymes or antibodies, with the sensitivity of electrochemical transducers. This integration facilitates the incorporation of new signal transduction technologies into biosensors, enabling quick, sensitive and straightforward analytes interrogation such as drugs, vitamins, metal ions and emerging pollutants, as well as in vivo analyses [15]. Although less common, the recognition element can be a whole cell, as in cell-based electrochemical biosensors that use living cells as sensing receptors and transducers to detect and convert physical, chemical, and physiological signals into electrical ones, providing valuable information about how cells interact with their environment [16].

Among the most suggestive examples of nanosized particles with impressive versatile applications are silver nanoparticles (AgNPs). AgNPs have become a subject of intense research and innovation, driving advancements in multiple fields, they exhibit exceptional antibacterial, antiviral, and catalytic properties, being a valuable resource in various industries, including healthcare, electronics, agriculture, and environmental remediation. Their small size and high surface area-to-volume ratio make them ideal for a wide range of applications, from enhancing drug delivery systems to improving the efficiency of sensors and catalysts. AgNPs can be fabricated through a sustainable and environmentally friendly process that harnesses the bioactive compounds found in various plant extracts. The reduction in silver ions by the phytochemicals present in plants leads to the formation of nanoscale silver particles with unique properties and applications. AgNPs have become a subject of intense research and innovation, driving advancements in multiple fields. In this paper, we explore, in extension, the synthesis of Ag nanoparticles using plant extracts, highlighting its sustainable capacities, versatility and cost-effective qualities, as well as the sensing capabilities [17,18,19].

There are several physical or solid-state reactions occurring via GS of NPs. Thermal or laser dewetting, for instance, are both processes of green nanoparticle synthesis, each holding common or different advantages. Laser dewetting, for once, is a nanofabrication process that involves using lasers to induce the agglomeration, or melting of thin films or coatings to create nanostructures, including NPs. This method is fast and is preferred in applications which require precise control over nanoparticle characteristics. Thermal dewetting, on the other hand, offers the advantage of sustainability and cost effectiveness by using green affordable materials and avoiding the use of toxic chemicals. This process involves using heat to induce the agglomeration and formation of NPs from a thin film or coating of a sustainable material such as organic materials or plant extracts resulting in NPs with unique properties and controlled characteristics [20]. Eco-friendly NPs can also be obtained using ion beam methods. Ion irradiation can be used to induce or assist the chemical reactions involved in the synthesis of NPs. For example, ion irradiation of precursor materials or plant extracts can promote the reduction and nucleation, facilitating their formation in a controlled manner [21]. A common disadvantage of all these methods is represented by the possibility to be applied only at a large scale of fabrication.

Sensors and biosensors drive innovation across diverse industries, from healthcare and agriculture to electronics and industry. They enable precise diagnostics, continuous monitoring, and advancements in personalized medicine. Biosensors ensure safety, quality control, and contamination detection in food and beverage production. They optimize irrigation techniques and crop health assessment in agriculture. The automotive industry relies on sensors for safety, automation, and innovative driver assistance systems. Integrated sensors in electronics and wearables monitor fitness and health, enable gesture recognition, and enhance augmented reality experiences. Industrial applications use sensors to enhance manufacturing processes, minimize downtime, and increase operational efficiency. Sensors and biosensors offer solutions to critical challenges and ensure real-time data analytics and enhanced efficiency.

This review provides a brief overview of the low-cost plant-based GS of NPs and their utilization in the fabrication of optical and electrochemical biosensors, and it explores the various perspectives and techniques involved in the GS of metallic particles, with a focus on the use of plant metabolites in NP synthesis for biomedical applications.

## 2. Sensors and Biosensors

A biosensor typically consists of two connected components: a chemical recognition system (the receptor) and a physicochemical transducer. The receptor system detects and interprets the interaction between the analyte and receptor, and then transfers this information to the transducer, which produces a measurable signal. This signal can then be read and recorded by a measuring device [22]. Biosensors can be divided into two categories based on the detection method used: optical and electrochemical sensors. Optical biosensors are highly effective in providing valuable information about a sample, such as kinetic behavior, concentration, and molecular interaction. One of the key advantages of optical biosensors is that they avoid electrical or magnetic interference, making them highly accurate. Additionally, they are highly sensitive and can detect analytes even at very low concentrations such as attomolar and femtomolar levels. However, despite these advantages, electrochemical biosensors are still the most commonly used type of portable biosensor, mostly because they are easier to miniaturize [23].

**Figure 1:**
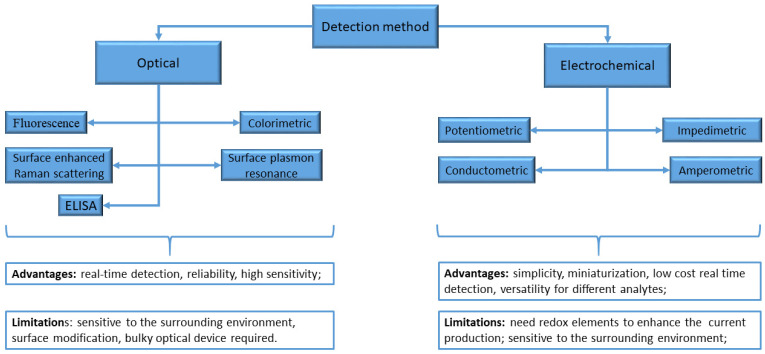
Biosensor categories based on the detection method.

Figure 1 shows the primary categories arranged according to their detection method [24].

Along with the notable progress in the development of nanoparticle-based biosensors, considerable opportunities for further enhancement have arisen. Among these, optimizing the dual functionality of nanoparticles, serving as both transducer components and recognition elements, stands prominent. The creation of multifunctional nanoparticles, including core–shell structures or composite materials, represents a significant advancement by integrating transduction properties with recognition elements, thereby amplifying biosensor performance. Ensuring stability and biocompatibility enhancements, such as employing surface coatings and biocompatible modifications, plays a pivotal role in sustaining functionality and mitigating interference within biological contexts. These systematic methodologies underscore the maximization of nanoparticle dual functionality, facilitating ongoing innovation in biosensor technology across diverse analytical domains encompassing healthcare, environmental monitoring, and beyond.

## 3. Green Synthesis of Metallic Nanoparticles from Plant Extract

In nanotechnology, it is crucial to develop reliable and eco-friendly methods to synthesize NPs. Conventional methods are either costly, toxic, not eco-friendly, or are not scalable. To address these issues, natural sources like plants are used to synthesize NPs. These sources act as reducing and capping agents. Green-synthesized MNPs have potential applications in drug delivery, DNA analysis, gene therapy, cancer treatment, antimicrobial agents, biosensors, catalysis, SERS, magnetic resonance imaging, and other new emerging fields. Over the past decade, interest has been growing in using plants to create NPs for biosensors. GS methods provide a sustainable and efficient approach to NP production, catering to the growing demand for environmentally friendly processes. GS methods are versatile, enabling the production of various types of NPs such as metal, metal oxide, and organic nanoparticles with enhanced stability and uniform size distribution, holding the necessary qualities for biomedical applications including biocompatibility. The use of plant extracts as reducing agents leads to achieving the controlled synthesis of NPs with distinct properties. Among the various methods of NP synthesis, GS, as mentioned before, has gained popularity due to its reduced environmental impact, energy efficiency, and potential for large-scale production.

The conventional and predominant techniques employed in synthesizing MNPs involve wet chemical processes which may sometimes offer cost-effectiveness for large-scale production, but come with disadvantages such as contamination from precursor chemicals, utilization of harmful solvents, and the generation of risky by-products. In a typical procedure, MNPs are cultivated within a liquid medium containing metal precursors and hazardous reactants as reducing agents, like sodium borohydride, potassium bitartrate, methoxy polyethylene glycol, or hydrazine. To prevent the clumping of MNPs, a stabilizing agent such as sodium dodecyl benzyl sulfate or polyvinyl pyrrolidone is added to the reaction mixture. In change, the biological approach to MNP synthesis comes with several notable advantages; natural resources, such as plants, can reduce inorganic metal ions to MNPs employing their own cellular metabolites, generating NPs with controlled shapes and sizes [25,26]. In plant extracts, there is a high number and quantity of bioactive compounds such as phytochemicals, polyphenols, flavonoids (e.g., quercetin, catechin), tannins, terpenoids, alkaloids and more, which often benefit human health, also holding the ability to utilize their multiple hydroxyl groups that can donate electrons to reduce metal ions and stabilize MNPs. Some examples of GS using redox active molecules of plant extracts can be provided.

The miscellaneous aqueous extract of green tea leaves with silver nitrate (AgNO_3_), at room temperature (RT), led to the formation of steady-state AgNPs due to the properties of tea polyphenolics in reducing Ag^+^ ions and stabilizing the resulted nanoparticles [27]. Based on the same principle, the active molecules of Aloe vera extract effectively reduced Au^3+^ from chloroauric acid (HAuCl_4_), with the formation of stable AuNPs [28,29,30].

In addition to the more well-known phenolic compounds with redox capacity presented in plants, terpenoids are a diverse class of compounds with similar properties, i.e., serving as both reducing and stabilizing agents in NP formation. Terpenoids like geraniol, limonene, and linalool contain multiple double bonds and functional groups, contributing to their reducing potential [31,32]. Alkaloids are nitrogen-containing compounds with diverse biological activities. Some alkaloids, such as those found in tea leaves (e.g., caffeine, theobromine, and theacrine) or in various medicinal plants can act as reduction agents during NP formation [33,34]. Sugars and carbohydrates present in plant extracts can participate in the reduction process due to their ability to donate electrons from hydroxyl groups. For instance, glucose, fructose, and sucrose are common carbohydrates that can serve as reducing agents [30,35]. Certain amino acids with reducing properties can also play a role in NP synthesis. Cysteine and histidine, for example, have functional groups that can donate electrons [36]. Some vitamins found in plant extracts, such as ascorbic acid (vitamin C), can act as effective reducing agents due to their electron-donating capabilities [37,38]. Enzymes can also contribute to the reduction process. Enzymes like reductases can facilitate the transfer of electrons and promote NP formation [39,40]. It must be mentioned that specific compounds present in a plant extract will depend on the plant species, its growth conditions, and the extraction methods used [41]. Different plants have varying combinations of these reducing agents, which can lead to differences in the efficiency and properties of the synthesized NPs.

**Figure 2:**
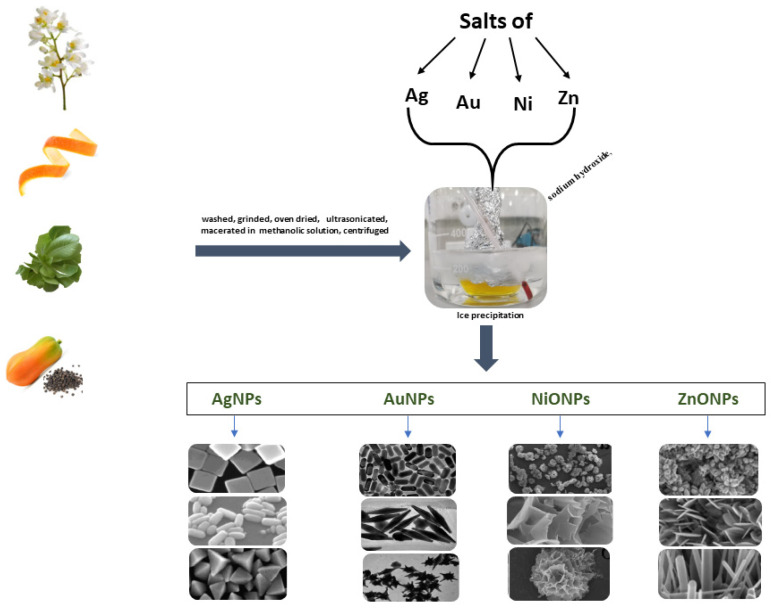
Examples of NPs synthetized using plant extracts AgNPs (reprint from [42]), AuNPs (reprint from [43]), NiONPs (reprint from [44]), and ZnONPs (reprint from [45]).

A schematic representation of plant-based green synthesis is given in Figure 2.

The utilization of green-synthesized nanoparticle-based sensors for genetic editing represents a prospective frontier in sensing technology. Gene editing methodologies currently occupy a preeminent position among biotechnological studies, carrying the imminent potential to profoundly influence various facets of human existence, encompassing health, longevity, nutrition, and numerous other domains [46]. Gene editing techniques, including CRISPR-Cas9 and other precise genetic modifications, offer unprecedented precision in altering the genetic makeup of biological receptors. In conjunction with sensing platforms, genetic editing allows for the targeted enhancements of receptor structures, augmenting their affinity and specificity toward diverse substances, even at low concentrations [47]. Nanoparticles, owing to their unique physicochemical properties, bolster the functionality of receptors, enhancing sensitivity, selectivity, and signal output in molecular detection processes. Moreover, green-synthetized nanoparticles, with their eco-friendly nature, align with sustainable practices, while their tailored characteristics facilitate efficient gene delivery and cellular targeting, enabling precise discrimination among various analytes. Moreover, the cost-effectiveness and versatility in functionalization makes green-synthetized nanoparticles important tools for contributing to a faster propulsion in genetic science research [48,49,50]. Apart from facilitating the detection of even traces amounts of substances, the science of genetic editing, coupled with the development of nanoparticle-based sensing platforms, also permits simultaneous analysis of multiple substances, achieving multiplexed sensing with exceptional accuracy [51,52]. This convergence of advanced techniques holds significant promise in reshaping the landscape of analytical sciences, fostering innovative solutions in environmental monitoring, healthcare diagnostics, biomedical research, food quality, and safety [53].

The present review paper explores this topic in depth. Basically, the plant-based GS of NPs is a straightforward procedure which requires only mixing at an optimal concentration of the metal precursors (such as silver, gold, and zinc salts) and a natural reducing agent taken from various leaves, fruits, or seeds.

## 4. Metallic Nanoparticle-Based Sensors

Noble metal nanoparticles make excellent structures for creating sensing devices due to their high surface-to-volume ratio. Their unique optical and electrical properties are very sensitive to environmental changes. These features ensure that sensing processes are highly sensitive.

**Figure 3:**
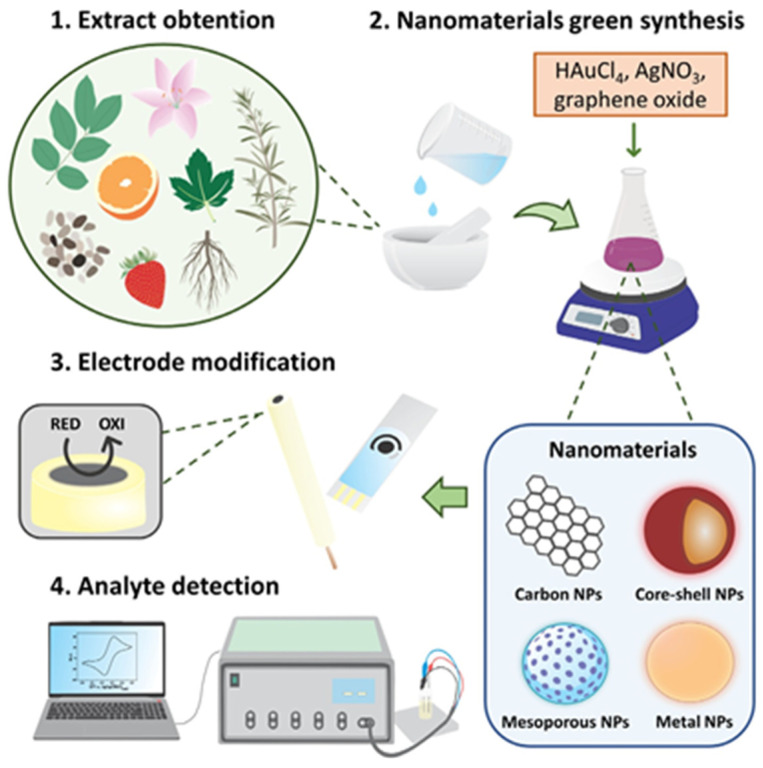
A general scheme of the biosynthesis of nanomaterials using vegetal samples, and their immobilization in electrochemical transducers for sensing applications. Reprint from [14].

Figure 3 illustrates a comprehensive schematic outlining the process of biosynthesizing nanomaterials using plant-based samples and subsequently immobilizing them in electrochemical transducers for various sensing applications. This overall approach is characterized by its simplicity, cost-effectiveness, and its potential to significantly enhance sensor construction procedures and detection capabilities.

Colorimetric sensing techniques that utilize nanoparticles typically rely on changes in their optical properties due to aggregation, dispersion, and changes in morphology. These modifications can result in plasmon resonance wavelength shifts, which can be measured to differentiate between various analytes [54,55]. The use of environmentally friendly materials, particularly plant extracts, for the synthesis of MNPs showed numerous benefits in pharmaceutical and other biomedical applications. The GS method was also very cost-effective and can be used as a cheap suitable alternative for the large-scale production of MNPs.

### 4.1. Ag Nanoparticles

The green route is the preferred method for synthesizing Ag nanoparticles due to its economic, non-toxic, fast, one-step, and environmentally safe process. Plant extracts are a valuable resource due to their easy availability and ability to produce safe and cost-effective non-toxic nanoparticles. For example, they can absorb metal ions from their surface or through roots and transport them to different organs and tissues, where reduction can occur. The effectiveness of a plant extract in synthesizing nanoparticles depends on the active compound that reduces Ag^+^ ions to Ag^0^, which can vary depending on the plant extract used. Moreover, plants possess an inherent ability to produce a diverse range of antioxidants that can counteract oxidative damage caused by reactive oxygen species (ROS). These antioxidants are composed of various organic molecules, including carbohydrates, fats, proteins, enzymes, coenzymes, phenols, flavonoids, terpenes, alkaloids, and more. These organic molecules can also be used to create silver nanoparticles through the reduction of silver nitrate (AgNO_3_) [56].

#### 4.1.1. Optical (Bio)Sensors Based on Ag Nanoparticles

**Figure 4:**
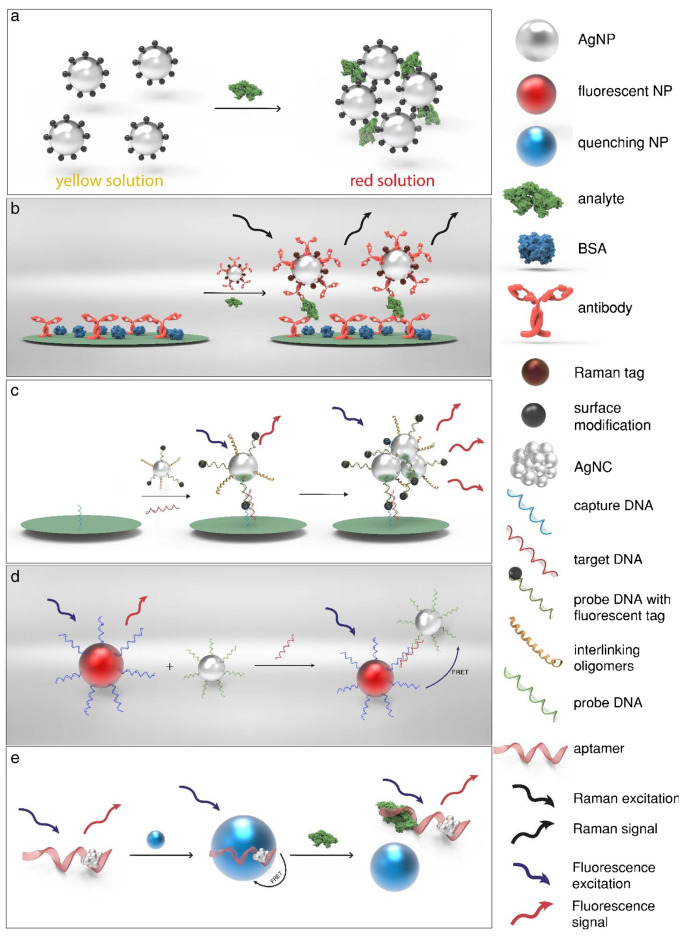
Schematic representation of the working principle of various optical biosensors using AgNPs based on (**a**) SPR, (**b**) SERS, (**c**) MEF, (**d**) FRET, or (**e**) the inherent fluorescent properties of AgNCs. Reprint from [57].

In recent years, there has been extensive research and development on optical sensors that use AgNPs, shown in Figure 4. The main difference between these sensors is the type of stabilizing agent used. These agents help protect the nanoparticle core and enable the nanoparticles to interact with specific analytes selectively. Using plant extracts to synthesize AgNPs is an eco-friendly method that has gained popularity as a green alternative.

In general, AgNP colorimetric sensors work by detecting changes in optical absorption when they come into contact with specific molecules. This gradual change in optical properties can be used to create an effective optical sensor that detects the analyte level. The sensitivity of these sensors depends on the functional group responsible for changing the properties of the AgNPs, which in turn alters the observed surface plasmon resonance (SPR) intensity, energy, and band shape. This allows for an accurate quantification of target molecules. Several examples for the use of AgNPs in optical sensing are as follows:

1. Detection of Hg^2+^ as a water pollutant by AgNPs.

It is essential to prioritize the protection of the environment and monitoring water quality to prevent potential health hazards caused by water contamination. Numerous research studies have demonstrated that metal nanoparticles can be integrated into sensors to detect water contaminants, such as heavy metal ions and pesticides [58]. Colorimetric sensors are a great option for detecting water pollutants due to their simplicity and affordability. These sensors can quickly determine contaminant concentrations and allow for on-site testing without complex equipment, making them a convenient and practical choice.

In this section, we highlight some studies that demonstrate this capability, as shown in Table 1. One example of this technology in action is the detection of Hg^2+^ (a common water pollutant) using AgNPs produced from various plant extracts.

Farhadi et al. has developed a method for detecting Hg^2+^ in aqueous environmental samples, using green-synthesized and unmodified AgNPs as a highly selective Hg^2+^ colorimetric sensor based on a redox reaction between AgNPs and Hg^2+^ present in the solution [59]. First, they developed an affordable and straightforward method of producing stable AgNPs using a bio-reduction process. This involved using a solution of AgNO_3_ and manna of Hedysarum aqueous extract as the reducing agent, along with soap-root extract as the stabilizer. It was easily observed with the naked eye that the yellowish-brown AgNP fresh solution was turned colorless in the presence of Hg^2+^, accompanying the broadening and blue shifting of the SPR band. This method has been tested with different metal ions and has proven highly selective for Hg^2+^ over other metals, with a detection limit of 2.2 × 10^−6^ M [60].

Another simple and low-cost method for detecting Hg^2+^ ions is using Agaricus Bispores (AB) to prepare AgNP–AB hybrids using a microwave reactor [61]. In an aqueous medium, the addition of Hg ions causes AgNP–AB to aggregate and change color from brown to black, resulting in the formation of an AgNP–AB–Hg complex. The electrochemical extended studies have shown that AgNP has metal sensing capabilities. Using a platinum electrode modified with the AgNP–AB highly sensitive sensor for the fast detection of toxic Hg ions, with a detection limit of 2.1 × 10^−6^ M, was achieved. Using Brassica oleracea var. botrytis (cauliflower-CLW) waste extract, the same microwave-assisted method was performed for the biosynthesis of AgNP–CLW to produce a Hg^2+^ biosensor [62]. In this framework, a visible color change from brown to pale yellow was observed when the synthesized AgNP–CWs were added to the solutions of Hg^2+^ containing 20.0, 1.0, and 0.1 mg/L. They also noted a decrease in NP SPR absorption, with the band intensity at a 411 nm wavelength. Their biosensing studies demonstrated that AgNP–CWs could specifically detect up to 0.1 mg/L Hg^2+^ ions.

Using hand-drawn paper, a colorimetric device for the detection of Hg^2+^ has also been developed [63]. The approach is selective and can be detected by the naked eye. This system employs a distinct method of sensor development through crayons to create hydrophobic barriers and a paper puncher to create patterns in the sensing area. The researchers prepared AgNPs by extracting Achillea Wilhelmsii (Aw), and tested their ability to detect Hg^2+^ in liquid and paper-based substrates using colorimetric methods. In the paper-based sensor, the target is quantified through a visual readout of a color-changed sensing zone modified with Aw–AgNPs. The color of Aw–AgNPs changes from brown to colorless in water and on paper when the target is present, with a detection limit of 28 × 10^−9^ M and 0.30 × 10^−6^ M for the two substrates, respectively, under optimal conditions.

Another colorimetric sensor based on AgNPs for detecting Hg^2+^ was derived from various leaf extracts. Recently, stable and spherical AgNPs using Acacia chundra leaf extract as the reducing and capping agents were synthesized, and a colorimetric sensor for Hg^2+^ in real water samples was developed [64]. When the synthesized AgNP solution and Hg^2+^ first interacted, the color of the detection reaction changed from yellow to reddish-brown, which was visible to the naked eye and it was found that the surface of the combined AgNPs had a high selectivity for Hg^2+^ ions (0.28 μM). In 2021, researchers used Citrus japonica (CJ) leaf extract as a biological liquid to synthesize stable AgNPs from silver salt [65]. The CJ–AgNPs proved to be highly sensitive, selective, stable, cost-effective, and eco-friendly, and a rapid colorimetric sensor for Hg^2+^ was obtained. The solution changed color from yellow to brownish upon detection. This sensor successfully detected low levels of Hg^2+^ (0.09 µM) in real water samples. In the same year, another group developed a method to synthesize green AgNPs using Mimosa diplotricha leaf extract (AgNP-MD). These NPs were found to be effective in detecting Hg^2+^ ions in an aqueous solution, serving as both optical and electrochemical sensors. The interaction between AgNP–MD and mercuric ions causes a color change from colored to colorless, indicating optical sensing. This change was due to the decreased SPR of the NPs. The team used cyclic voltammetry (CV) and differential pulse voltammetry (DPV) to study the AgNP–MD–Pt electrode electrochemical sensing of Hg^2+^ ions. A redox couple, in CV, showed the interaction between Hg^2+^ ions and AgNP–MD on the Pt electrode surface. The DPV response of the AgNP–MD–Pt electrode toward various concentrations of Hg^2+^ ranging from 5 to 45 μM yielded a limit of detection (LOD) value of 1.46 µM [66].

A straightforward and eco-friendly method of creating AgNPs using the bioactive components at molasses, such as reducing, capping, and stabilizing agents, at room temperature (RT), has been successfully developed for detecting Hg^2+^ ions with high selectivity and sensitivity via colorimetric analysis, with a minimum detection limit of 0.025 µM in real water bodies [67].

2. Detection of ammonia by AgNPs

Ammonia is a versatile substance used in various industries such as fertilizer, animal feed, fiber, plastic, paper, pharmaceutical, and explosives. On the other hand, ammonium salts are utilized as food additives and cleansing agents. It is important to note that ammonia and its salts are both hazardous and caustic, which can pose serious health risks to humans, fish, and crustaceans. Aquatic species, in particular, excrete ammonia and cannot convert it to less toxic compounds, making them susceptible to toxic effects at high concentrations. Therefore, it is crucial to detect, sense, and monitor ammonia levels in water. Optical sensors for the detection of dissolved ammonia were made through the GS of AgNPs using the aqueous fruit extract of Terminalia chebula [68], polysaccharide Cyamopsis tetragonaloba (guar gum) [69], and sugarcane leaf extract [70]. The process involved the formation of AgNPs, confirmed by observing a color change in ammonia-containing solutions due to the formation of an AgNP–ammonia complex which amplified and shifted the SPR absorbance intensity. When the polysaccharide Cyamopsis tetragonaloba was utilized, a detection limit of 1 ppm was determined for the ammonia solution.

3. Detection of different heavy metal ions as water pollutants by AgNPs

Green-synthesized silver nanoparticles can detect other hazardous heavy metal ions such as Cu^4+^, Cd^2+^, and Cr^3+^ in water. These toxic metals pose a threat to human life and health. A complete colorimetric sensor was developed based on lignin-stabilized AgNPs from Acacia wood to evaluate the sensing of different heavy metal ions over the broad range between 1 nM to 100 mM [71]. The absorption peak shifts due to the reduction in metal ions by AgNP, and the subsequent deposition of elementary metal on the surface of nanoparticles. Also, AgNPs were created using various plant extracts to detect copper, chromium, and cadmium ions in water. Specifically, AgNPs were synthesized using Moringa oleifera flower extract to detect copper ions, with a detection limit of 0.249 mM [72]. Researchers used Allium sativum extract to detect cadmium ions to create AgNP without any surface functionalization [73]. Using DPV, they calculated the system’s detection limit to 0.277 μM. Another group developed a new type of sensor that uses AgNPs from Lycopersicon esculentum extract to detect Cr ions [74]. The linear calibration range, based on DPV results for this system was between 10 to 90 μM, and the detection limit was found to be 0.804 μM.

4. Detection of H_2_O_2_ by AgNPs

Green-synthesized AgNPs have the potential to determine the presence of hydrogen peroxide (H_2_O_2_), which is relevant in environmental, pharmaceutical, and clinical research. H_2_O_2_ is a powerful oxidizing agent produced as a byproduct of enzymatic reactions involving glucose, cholesterol, and lactate. However, excessive H_2_O_2_ can lead to serious health issues, and it is crucial to develop a reliable method for detecting concentrations as low as possible, maintaining, at the same time, a broad linear range. The H_2_O_2_ sensing technology has also continuously been developed in the same manner as ammonia regarding environmental and health industries. An innovative dual colorimetric sensor was developed by Srikhao et al. to detect both ammonia and H_2_O_2_ [70]. Their study involved the use of AgNO_3_ as a precursor and sugarcane leaf extract as reducing agents to perform the green synthesis of AgNPs. The results showed that the silver nanoparticles could detect hydrogen peroxide and ammonia at low concentrations of 30 mM and 5 ppm, respectively. A polysaccharide called locust bean gum was extracted from Ceratonia siliqua and used as a stabilizing and reducing agent to create AgNPs. These nanoparticles were employed to develop an optical fiber-based sensor that was able to detect concentrations of H_2_O_2_ as low as 0.01 mM [75].

A colorimetric sensor using green algae has been developed using Noctiluca scintillas extract as a nanoparticle capping shell. The plant extract has a self-reducing activity that converts silver ions to metal nanosilver without toxic reducing agents like NaBH_4_. This system has been proven to be a reliable colorimetric sensor for detecting H_2_O_2_. It has the lowest detection limit for H_2_O_2_ compared to other species, and the test showed a color change from brown to colorless, with H_2_O_2_ presenting the most noticeable change in color. The test assay provides accurate and reproducible values of H_2_O_2_ in unknown samples in three different ranges (nM, µM, and mM) using ΔAbs calibration curves [76].

The plant extracts Justicia adhatoda L. (JALE) and Atalantia (A) monophyla were utilized to create AgNPs that can detect H_2_O_2_. The green-synthesized JA–AgNPs were particularly effective in detecting reactive oxygen species (ROS) in biological samples and waterbodies. They showed exceptional sensing abilities and could detect H_2_O_2_ based on a colorimetric response. On the other hand, AgNP-A. monophyla systems also detected H_2_O_2_ by observing a decrease in absorbance and the disappearance of the silver SPR peak at 340 nm. Overall, these findings suggest that these AgNPs could be useful for detecting H_2_O_2_ in various settings [77,78]. Based on the same principle, another group developed a colorimetric sensor to detect H_2_O_2_ using a green-synthesized system made of polylactic acid (PLA)/silver nanoparticles (PLA–AgNPs). By observing the fading color of the solution and the disappearance of the characteristic SPR absorbance peak, they can accurately detect even very low concentrations of H_2_O_2_ (5 μM). This sensor is useful for biological and environmental analysis, as it was created by reducing AgNO_3_ to nanosilver (n-Ag) using Piper nigrum leaves extract in a dual-phase medium containing PLA (capping agent) [79].

#### 4.1.2. Ag-Based Electrochemical (Bio)Sensors for Pharmaceutical and Bioactive Molecules

Electrochemical methods are popular in metal sensing due to their low cost, ease of use, and selectivity. This technique involves modifying an electrode with a ligand or chelating agent to selectively detect metal ions. Various sensors, such as magnetic nanoparticles, nanoclusters, and metal nanoparticles, have been used as carriers to obtain electrochemical signals. Among these NPs, AgNPs are commonly used for the surface modification of electrodes due to their low cost and excellent electrical and physio-chemical properties, characterized by high electron transfer rates and low detection limits [72].

Several methods and morphologies have been reported for AgNP-modified graphene oxide nanocomposites, owing to their exceptional electrocatalytic properties for H_2_O_2_ sensing. In this context, silver nanoparticle-modified reduced graphene oxide nanocomposites (rGox/AgNPs) were obtained using the GS method which involved tea and Andrographis paniculata extracts to reduce both Ag^+^ cations and graphene oxide sheets [80]. The tea polyphenols acted as an efficient and soluble reducing agent. The glassy carbon (GC) electrodes were modified with the rGox/AgNPs nanocomposite, and the electrochemical tests were performed to evaluate the electrocatalytic properties of the resulting GCE/rGox/AgNPs sensor against H_2_O_2_ reduction. For a concentration range of 0.002 to 20 mM H_2_O_2_, the sensitivity of 236 μA mM^−1^ cm^−2^ (R^2^ = 0.999), the response time of 2 s, and a detection limit of about 0.73 μM were achieved. A Ag–GO nanocomposite was also achieved using Andrographis aniculate extract as the reducing agent, which exhibited excellent sensing ability for H_2_O_2_, with a low detection limit of 2.65 μL and a wide detection range of 0–15 μM [81].

Various research studies have reported electrochemical sensors that utilize modified GC electrodes with green-synthesized AgNPs to detect biomolecules of interest. Recently, *C. sempervirens* pollen extract was utilized to produce AgNPs in an environmentally friendly manner. A GC electrode was modified with the obtained AgNPs to create AgNP–GCE, which was tested on H_2_O_2_, and a detection limit of 0.23 μM was obtained [82]. In other studies, a GC modified with AgNPs biohybrids, obtained using Araucaria angustifolia pine nuts and exfoliated graphite nanoplatelets, was employed for the detection of paracetamol, and an LOD of 8.50 × 10^−8^ M was obtained [83].

Bisphenol A (BPA) is a significant substance used as a plasticizer in producing epoxy resins and polycarbonate plastics. Several studies have shown that BPA migrates from plastic consumer products into the human body, as well as from industrial effluents into environmental samples, resulting in multiple health and environmental concerns. To address the issues, electrochemical biosensors were developed to detect BPA using AgNPs synthesized using green methods. An example is a straightforward electrochemical sensor platform that uses a composite of functionalized multi-walled carbon nanotube (f-MWCNT) and AgNPs synthesized from Cinnamomum tamala extract (AgNps/f-MWCNT) [84,85]. This sensor can detect a wide range of concentrations, from 3.9 fM to 102.4 nM, with a low detection limit of 0.38 nM. A similar sensor was developed using Moringa oleifera extract for the GS of AgNPs, f-MWCNT as catalysts, and GC electrode surface as the transducer [86]. By the means of square-wave voltammetry, an LOD of about 0.22 µM was obtained for a linear range of 0.3 to 8 µM.

### 4.2. Au Nanoparticles

Gold nanoparticles (AuNPs) are becoming increasingly popular due to their potential uses in areas such as electrochemical, catalysis, and optical sensors. The use of plants to obtain these NPs using GS methods has many advantages over traditional chemical and physical methods. AuNPs have multiple surface functionalities that make them highly versatile and adaptable when used with various biological assemblies or modifications, resulting in improved applications. This eco-friendly approach is easily scalable and does not require high pressures, energy-intensive environments, high temperatures, or toxic chemicals. Plants are widely available and safe to handle, making this synthesis method a promising alternative to conventional techniques. This procedure has the added benefit of being safe to handle, widely available, and can be developed using conventional ideas [87,88].

#### 4.2.1. Optical Sensors Based on Au Nanoparticles

**Figure 5:**
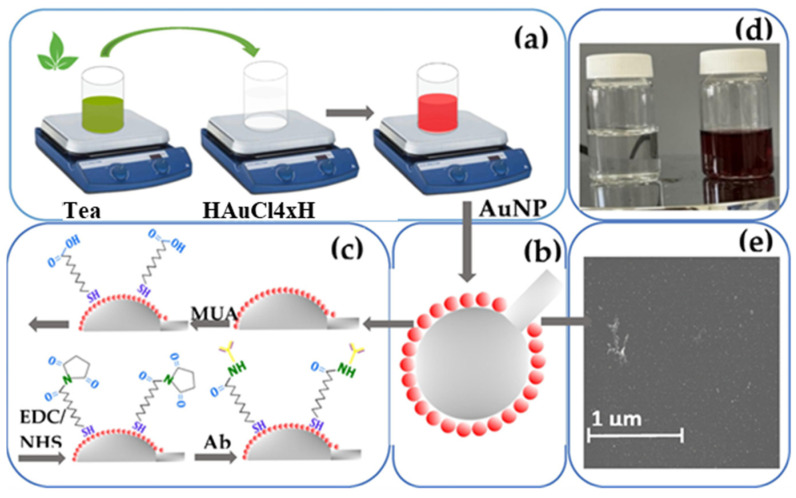
Schematic overview of the full functionalization of ball resonator optical fibers using green-synthesized AuNPs decorated with biological recognition elements to detect an analyte: (**a**) the synthesis of AuNPs from green tea extract; (**b**) the immobilization of AuNPs onto the surface of the ball resonator optical fiber after the APTMS treatment; (**c**) the functionalization of the gold-coated sensor with antibody after pre-treatment with MUA and EDC/NHS; (**d**) the visual change in color from colorless to wine-red during synthesis; (**e**) the SEM image showing the surface of ball resonator optical fibers attached to AuNPs. Reprint from [92].

An optical sensor that can detect aqueous ammonia has been created using guar gum (GG) as a reducing agent to produce AuNPs via GS [89]. This method has proven highly reproducible, with response times of approximately 10 s and exceptional sensitivity, detecting concentrations as low as 1 ppb. Another plant-derived polysaccharide from Gum Karaya was used as a reducing, stabilizing, and functionalizing agent to obtain green AuNPs. These nanoparticles change color from red to blue when exposed to copper ions, making them useful for detecting copper in real water samples and biological samples. The detection system demonstrated a strong linear correlation (R^2^ = 0.998) for a linear range between 10 and 1000 nM of Cu^2+^, with a detection limit of 10 nM [90]. A colorimetric sensor was used to detect cysteine, a light aminothiol found in human plasma [91]. This sensor was based on AuNPs obtained from the willow tree bark extract. The interaction between the synthesized nanoparticles and cysteine caused a change in the SPR band, resulting in a purple color change. The intensity of the color change was proportional to the cysteine concentration within a specific range. A recent study has introduced a biosensing platform for detecting the CD44 cancer biomarker using AuNPs obtained via green tea leaf synthesis, as presented in Figure 5 [92].

The platform was built on the surface of ball resonator optical fibers. The investigation showed that the sensitivity of the ball resonator optical fiber biosensor increased rapidly, reaching a maximum of 13.17 dB in intensity change. This increase occurred with each 10-fold concentration increase, ranging from 42.9 aM to 100 nM. The study also evaluated the detection of CD44 antigen at various concentrations and determined the LOD to be 0.111 pM. Green tea extracts were also involved in synthesizing AuNP for the colorimetric detection of acetamiprid in a SERS-based biosensor design [93]. In the presence of acetamiprid, the SERS intensity increased linearly from 30 nM to 4.0 µM, with a relatively low limit of 17.6 nM.

#### 4.2.2. Electrochemical Biosensor Based on Au Nanoparticles

An electrochemical detection system for accurately measuring L-tryptophan (Trp) levels in biological samples was developed using a screen-printed electrode surface modified with a biohybrid consisting of a rGO/AuNPs assembly synthetized using E. tereticornis leaves as the sustainable reducing agent [94]. Under optimal conditions, the LOD and limit of quantification (LOQ) were 0.39 and 1.32 µM, respectively, for a linear range between 0.5 and 500 M. Another study addressing glucose detection at rGO/AuNPs, using Rose water as a reducing agent, reported an LOD of 10 μM for a linear range of 1 to 8 mM [95].

The amperometric determination of “hlor”phenicol in milk, powdered milk, honey, and eye drops based on the use of AuNPs/GO was obtained using Bischofia javanica Blume leaves as the reducing agent for the synthesis of the nanoparticle assembly [96]. The amperometric sensing platform had a broad linear range of 1.5–2.95 μM, a low detection limit of 0.25 μM, and high sensitivity of 3.81 μA μM^−1^ cm^−2^ under optimal conditions. The sensor also displayed reliable repeatability, reproducibility, anti-interference ability, and long-term storage stability.

Using another approach, the AuNPs prepared via GS from Sargassum were used to modify a carbon nanotube -screen-printed electrode using the drop-casting method, and to assemble a novel portable electrochemical sensing platform for small concentrations of glucose (50 µM) [97].

### 4.3. Non-Noble Metals

#### 4.3.1. Ni and NiO Nanoparticles

Numerous studies have emphasized the versatility of nickel nanoparticles (NiNPs) and nickel oxide nanoparticles (NiONPs) in various fields, such as environmental remediation, microbiology sensing, and biosensing. Because the rapid detection of glucose plays a crucial role in the diagnosis and management of diabetes, and a large segment of the population is affected by it, intensive attention was oriented toward one important application of NiONPs, which involves glucose sensing detection.

In view of all the above, it is important to mention that several studies that have been carried on the subject. For instance, in a recent study, in 2022, a non-enzymatic sensor for glucose determination was developed utilizing a GC electrode modified with NiONPs obtained via GS based on Nigella sativa extract [98]. The modified electrode showed a well-defined redox couple of Ni^3+^/Ni^2+^, indicating the efficient electrogeneration of Ni^3+^ species on the electrode surface, and demonstrating effectiveness of detection. The glucose detection exhibited two linear ranges of detection: one from 50 µM to 600 µM and another from 1 mM to 10 mM. For both ranges, a time response of about 3 s, an LOD of approximately 3.2 µM, and a sensitivity of 987.85 mA cm^−2^ mM^−1^ in the lower range and 170.85 mA cm^−2^ mM^−1^ in the higher range, were obtained. A similar electrochemical sensing platform, for glucose detection, was proposed in a study based on the GS of NiONPs using an aqueous extract of Trigonella subenervis (TS) [99]. The NiONP–TS exhibited a uniform spherical morphology with a size of 28.21 nm according to FE-SEM (field effect scanning electron microscopy) images, while XRD (X-ray diffraction) analysis revealed a crystallite size of 26.43 nm, and the electrochemical investigation demonstrated significant electrocatalytic activity for glucose oxidation. The sensor exhibited a linear response range of 10–200 μM and a detection limit of 3.2 μM, highlighting its potential as a glucose-sensing platform.

Taking advantage of the sponge-like structure of pomelo peel and its ability to be loaded with a large quantity of Ni^2+^ ions, graphene foam-like 3D porous carbon/NiNP nanocomposites were fabricated using a carbonization process [100]. Extensive characterization via SEM, TEM, N_2_ adsorption/desorption isotherms, XRD, XPS, and Raman spectra, as well as electrochemical techniques, revealed that the NiNPs acted as a catalyst, facilitating the transformation of the sponge-like pomelo peel into the graphene foam-like 3D porous carbon/NiNPs nanocomposites during the carbonization process. These nanocomposites exhibited unique catalytic activity, and good electrical conductivity, contributing to the development of an electrochemical sensor for glucose detection. The sensor displayed a wide linear range of 15.84 μM to 6.48 mM and a low detection limit of 4.8 μM.

Using a microwave-assisted method and Tagetes erecta L leaf extract as a bio-reductor, NiONPs, sized around 18.2 nm and having efficient photocatalytic activity (degradation efficiency of 53% for 120 min) and excellent electrochemical sensing capabilities for glucose detection, were synthesized [101]. The detection limit for glucose was less than 83 µM, and a sensitivity of 2556.41 µAmM^−1^ cm^−2^ was achieved. Additionally, these NPs exhibited significant antibacterial activity against both Gram-positive and Gram-negative bacterial strains, showing superior efficacy against Gram-positive strains.

Besides glucose sensing, green-synthesized NiNPs expressed their utility for several other sensing applications. For instance, a non-enzymatic method for the electroanalytical detection of nitrite was developed [102]. The employed solution used the combustion method to produce nickel oxide nanoparticles using an extract derived from C. gigantea leaves as the reducing agent. The synthesized NiONPs were characterized using various analytical techniques, demonstrating the presence of rhombohedral-structured crystallites, with an average size of 31 nm. The nanoparticles had a bandgap of 3.45 eV and possessed photodegradation abilities against methylene blue. Being employed as a non-enzymatic nitrite sensor, these NPs were able to demonstrate a wide linearity range of 8–1700 μM and exhibited good stability, with an LOD of 1.2 μM.

Hybrid nanocomposites containing bicomponent nanoparticles, such as Ni/Ag and GO can also be obtained using GS. Such structures were obtained using Punica granatum (pomegranate) juice and were employed in fabricating a sensor for the detection of ascorbic acid [103]. The electrochemical impedance spectroscopy revealed a 30.0 Ohm resistance for the sensor. For a linear range of 4.89–90.09 µM, an LOD of 0.16 µM, and a sensitivity of 23,381 µA cm^−2^ mM^−1^, for ascorbic acid, were obtained. Interference studies indicated that this sensor manifested selectivity toward detecting ascorbic acid. Furthermore, it demonstrated excellent stability for up to 45 days, showcasing its long-lasting performance.

#### 4.3.2. Zn and ZnO Nanoparticles

Zinc nanoparticles (ZnNPs) and zinc oxide nanoparticles (ZnONPs) have garnered significant attention in various scientific disciplines due to their unique physical and chemical properties. Different studies highlighting the fact that ZnNPs exhibit unique optical, electrical, and catalytic properties, making them highly desirable for applications in diverse fields such as electronics, photonics, catalysis, biomedicine, and environmental remediation [104,105]. Similar to other NPs, ZnNPs have garnered significant attention due to their capacity to be synthesized using plant extracts. It is well known that the reducing and stabilizing properties of phytochemicals present in plant extracts might facilitate the formation of NPs, and it was proved that high surface area-to-volume ratio, tunable reactivity, and compatibility with biological systems make them suitable for various applications [106]. The properties of ZnNPs can be tailored by controlling various synthesis parameters, including the type of plant extract used, reaction conditions, and post-synthesis treatments. Incorporated in sensing platforms, ZnNPs and ZnONPs become of great use as part of various types of sensors and (bio)sensors, including glucose and silymarin biosensors, formaldehyde electrochemical sensors, ethanol vapor sensors, carbon monoxide sensors, and as per exemplification, few research studies are called for attention in this review.

Combining the catalytical properties of NPs with the selectivity of enzymes, highly sensitive and stable biosensors can be obtained. Starting from the GC electrode as a transducer, using the peach extract for the synthesis of hybrid ZnONP–nitrogen-doped carbon sheets, and glucose oxidase as a recognition element, a biosensor for glucose detection was proposed. For a broad linear range, 0.2–12 mM, an LOD of 6.3 μM, and a sensitivity of 231.7 μA mM^−1^ cm^−2^ were obtained [107].

Other hybrid systems such as ZnO, carbon-based nanostructure were also explored. Based on GS using Carica papaya seed extract, ZnONPs, with the size ranging from 4 to 8 nm, were obtained and combined with multiwalled carbon nanotubes at the surface of a GC electrode [108]. The sensing capabilities toward silymarin were investigated, and a 76% detection efficiency, in a commercial Milk Thistle tablet was obtained. The electrochemical study was accomplished by the density functional theory calculations indicating that functional groups in silymarin facilitate the binding with ZnONPs and subsequent detection.

Citrus sinensis peel waste was used to fabricate ZnONPs assembled in a low-cost device, based on graphite paste electrode, for the electrochemical detection of formaldehyde [109]. After optimization, a high selectivity from ethanol was achieved, and a recovery rate up to 103.9%, measured in a tofu sample, with an RSD ratio value of 0.38, was obtained. For ethanol detection, the gas-sensing properties of ZnONPs, of 23 nm, synthesized using dye extract obtained from Ixora Coccinea leaves, were investigated [110].

A high-tech application in organic field-effect transistor (OFET) devices, for carbon monoxide (CO) gas sensing was developed based on the cost-effective fabrication of ZnONPs through a combustion synthesis method mediated by Nelumbo nucifera (lotus) leaf extract. The various characterization techniques demonstrated the formation of highly pure and crystalline ZnONPs with a nearly spherical shape and an average size of 3–4 nm. Further, a p-type organic field-effect transistor (OFET) device was fabricated using poly(3-hexylthiophene-2,5-diyl) (P3HT) and ZnONPs. The device exhibited field-effect mobility of 10^−2^ cm^2^ V^−1^ s^−1^ and emphasized a slightly enhanced response in detecting CO gas at RT, a fact confirmed by variations in key electrical parameters such as field-effect mobility (μ), on-current (Ion), and off-current (Ioff). Notably, the fabricated device exhibited superior selectivity and sensitivity in detecting CO gas compared to other reducing gases (H_2_S and NH_3_) and methanol vapors tested during the experiments [111].

Plant-based nanoparticles used in volatile organic compound (VOC) sensors offer numerous advantages, including sensitivity, selectivity, rapid response, portability, and versatile applications. These qualities position them as promising tools for detecting and monitoring VOCs across various industries. Nanoparticles provide a heightened surface area-to-volume ratio, enabling intensified interactions with VOCs. This characteristic allows for the detection of minute quantities and finds applications in environmental monitoring, such as assessing air quality, detecting pollutants, and tracking emissions, thereby showcasing heightened sensitivity [112]. Nanoparticle-based sensors can be integrated into miniaturized devices, enhancing portability and enabling on-site, point-of-care VOC detection with swift response times. This facilitates timely detection in crucial sectors like agriculture, healthcare, and other significant industries [113,114]. Functionalized nanoparticles enable targeted recognition and capture specific VOCs within complex matrices, ensuring precise and selective sensing capabilities. Such capabilities yield positive outcomes in healthcare diagnostics, such as breath analysis, and in industrial settings by ensuring workplace safety through the detection of hazardous VOCs [115].

**Table 1:** List of plant-based green-synthesized MNP systems as colorimetric and electrochemical sensors for the detection of different analytes: type of extract and sensing feature.

MNP Systems	Type of Extract	Detected Analyte	Linear Range	LOD	Reference
AgNP–Hedy-arum	*Hedysarum aqueous*; *Soap-root* *extract*	Hg(II)	10–100 µM	2.2 μM	[60]
AgNP–AB	*Agaricus Bispores*	Hg(II)	10–90 µM	2.1 μM	[61]
AgNP–CLW	*Cauliflower-Brassica oleracea* *var. botrytis*	Hg(II)		0.49 μM	[62]
AgNP–Aw	*Achillea Wilhelmsii*	Hg(II)	100 nM–100 µM 10–700 µM	28 nM on solution 0.3 μM on paper	[63]
AgNP–AC	*Acacia chundra*	Hg(II)		0.28 μM	[64]
AgNP–CJ	*Citrus japonica (CJ)*	Hg(II)	0.3–7.3 µM	0.09 μM	[65]
AgNP–MD	*Mimosa diplotricha*	Hg(II)	5–45 μM	1.46 μM	[66]
AgNP–M	*Molasses*	Hg(II)	0.01–1 μM	0.02 μM	[67]
AgNP–TC	*Terminalia chebula*	Ammonia	0–100 ppm	50 ppm	[68]
AgNP–GG	*Cyamopsis tetragonaloba*	Ammonia	1–50 ppm	1 ppm	[69]
AgNP–SG	*Sugarcane leaves*	H_2_O_2_	0–200 mM	30 mM	[70]
		Ammonia	0–50 ppm	5 ppm	
AgNP–MOF	*Moringa oleifera flower*	Cu(IV)	1–12 mM	0.249 mM	[71]
AgNP–AS	*Allium sativum*	Cd(II)	10–90 μM	0.277 μM	[73]
AgNP–LE	*Lycopersicon esculentum*	Cr(III)	10–90 μM	0.804 μM	[74]
AgNP–LBG	*Ceratonia siliqua*	H_2_O_2_	0.01–1 mM	0.01 mM	[75]
AgNP–Algae	*Noctiluca scintillans*	H_2_O_2_	4.70–32 nM	1.34 nM	[76]
AgNP–rGOx	*Tea*	H_2_O_2_	0.002–20 mM	0.73 μM	[80]
Ag–GO	*Andrographis paniculata*	H_2_O_2_	0–15 μM	2.65 μM	[81]
AgNPs–GCE	*C. sempervirens pollen*	H_2_O_2_	5 μM–2.5 mM	0.23 μM	[82]
AgNP–xGnP	*Araucaria angustifolia*	paracetamol	4.98 × 10^−6^–3.38 × 10^−5^ mol L^−1^	8.50 × 10^−8^ mol L^−1^	[83]
AgNps/f-MWCNT	*Cinnamomum tamala*	BPA	3.9 fM–102.4 nM	0.38 nM	[84]
AgNps/f-MWCNT	*Moringa oleifera extract*	BPA	0.3–8 µM	0.22 µM	[86]
AuNP–GG	*Guar Gum (GG)*	ammonia	-	1 ppb	[89]
AuNP–CMGK	*Gum Karaya*	Cu(II)	10–1000 nM	10 nM	[90]
AuNP–WTB	*Willow tree bark*	cysteine	2 × 10^−7^–20 × 10^−7^ mol/L	0.63 × 10^−7^ mol/L	[91]
AuNP–tea	*Green tea*	CD44 antigen	42.9 aM–100 nM	0.111 pM	[92]
AuNP–tea	*Green tea*	Acetamiprid	3.0 × 10^−8^–4.0 × 10^−6^ M	1.76 × 10^−8^ M	[93]
AuNP–rGO	*E. tereticornis*	L-tryptophan	0.5–500 µmol/L	0.39 µmol/L	[94]
AuNP–rGO	*Rose*	glucose	1–8 mM	10 µM	[95]
AuNP–GO	*Bischofia javanica Blume*	chloramphenicol	1.5–2.95 μM	0.25 μM	[96]
AuNP–CNT–SPE	*Sargassum* sp.	glucose	1–7 mM	50 µM	[97]
NiNP–NS	*Nigella sativa*	glucose	50–600 µM	3.2 µM	[98]
NiONP–TS	*Trigonella subenervis*	glucose	10–200 μM	3.2 µM	[99]
NiNP–PP	*Pomelo Peel*	glucose	15.84 μM–6.48 mM	4.8 µM	[100]
NiONP–TE	*Tagetes erecta* L.	glucose	0.1–1 mM	<83 µM	[101]
GCE/ZnO–NDCS/GOx	*peach juice*	glucose	0.2–12 mM	6.3 µM	[107]
GCE/MWCNTs/ZnO NPs	*Carica papaya*	Silymarin	0.014–0.152 mg/L	0.062 mg/L	[108]
GPE/ZnO NPs	*Citrus sinensis*	formaldehyde	0–100 mM	18 μM	[109]
ZnONPs	*Ixora Coccinea*	Ethanol	40–800 ppm	200 ppm	[111]

## 5. Conclusions

The aim of this review was to highlight the potential of plant-based synthetized nanoparticles to be used in the fabrication of sensors and biosensors with applications in different fields. The advantages of (bio)sensors in dynamic real-time applications compared to conventional assays highlight the potential of biosensors for an improved quality of life for all. These advantages include, but are not limited to, improved selectivity and sensitivity, high catalytic effect, low detection limits, etc.

The sensing and immobilization methodologies are important steps in biosensors fabrication. One of the candidates used either for immobilization or sensing is metallic nanoparticles. The use of plant extracts for the synthesis of metallic nanoparticles such as AgNPs, AuNPs, NiNPs, and ZnNPs has shown great promise. This green method requires non-toxic chemicals to reduce metallic salt. These nanoparticles have versatile properties and can be used to detect heavy metal ions in water as optical biosensors or pharmaceutical and bioactive molecules as electrochemical biosensors.

This review has examined the performances of different (bio)sensors which integrate plant extract synthetized NPs and their detection limits in various linear ranges. Thus, the present article highlighted the advances of this green methodology over the last ten years in the field of (bio)sensors.

Biosensing technologies have led to significant advancements in the development of innovative nanoparticles, which enable unprecedented levels of bio-chemical sensitivities that are critical for diseases and environmental monitoring. Additionally, miniaturized biosensors and their integration across large areas have allowed for high-density biosensing panels, which are essential for high throughput monitoring. By integrating biological and electronic elements, biosensors can provide rapid and precise results, allowing for real-time monitoring and high sensitivity. This offers the potential for swift and accurate health quality assessment. In order to achieve a new, emerging supra-disciplinary field that bridges production and extensive characterization with the prototyping of the most promising biosensor in realistic operative conditions, strong collaboration between researchers and the private sector is necessary.

## Data Availability

Data sharing not applicable.

## References

[1] Milone A., Monteduro A.G., Rizzato S., Leo A., Di Natale C., Kim S.S., Maruccio G. (2023). Advances in Materials and Technologies for Gas Sensing from Environmental and Food Monitoring to Breath Analysis. Adv. Sustain. Syst..

[2] Wasilewski T., Neubauer D., Kamysz W., Gębicki J. (2022). Recent progress in the development of peptide-based gas biosensors for environmental monitoring. Case Stud. Chem. Environ. Eng..

[3] Palumbo M., Attolico G., Capozzi V., Cozzolino R., Corvino A., de Chiara M.L.V., Pace B., Pelosi S., Ricci I., Romaniello R. (2022). Emerging postharvest technologies to enhance the shelf-life of fruit and vegetables: An overview. Foods.

[4] https://www.iso.org/home.html.

[5] https://www.astm.org/.

[6] Ealias A.M., Saravanakumar M.P. (2017). A review on the classification, characterisation, synthesis of nanoparticles and their application. IOP Conf. Ser. Mater. Sci. Eng..

[7] Pal G., Rai P., Pandey A. (2019). Green synthesis of nanoparticles: A greener approach for a cleaner future. In Green synthesis, characterization and applications of nanoparticles. Micro and Nano Technologies.

[8] Dutta D., Das B.M. (2021). Scope of green nanotechnology towards amalgamation of green chemistry for cleaner environment: A review on synthesis and applications of green nanoparticles. Environ. Nanotechnol. Monit. Manag..

[9] Patil S., Chandrasekaran R. (2020). Biogenic nanoparticles: A comprehensive perspective in synthesis, characterization, application and its challenges. J. Genet. Eng. Biotechnol..

[10] Das R.K., Pachapur V.L., Lonappan L., Naghdi M., Pulicharla R., Maiti S., Cledon M., Dalila L.M.A., Sarma S.J., Brar S.K. (2017). Biological synthesis of metallic nanoparticles: Plants, animals and microbial aspects. Nanotechnol. Environ. Eng..

[11] Dikshit P.K., Kumar J., Das A.K., Sadhu S., Sharma S., Singh S., Gupta P.K., Kim B.S. (2021). Green synthesis of metallic nanoparticles: Applications and limitations. Catalysts.

[12] Al-Tamimi S.A. (2021). Biogenic green synthesis of metal oxide nanoparticles using oat biomass for ultrasensitive modified polymeric sensors. Green Chem. Lett. Rev..

[13] Mandal D., Mishra S., Singh R.K. (2018). Green synthesized nanoparticles as potential nanosensors. Environmental, Chemical and Medical Sensors.

[14] Hacke A.C.M., Lima D., Kuss S. (2022). Green synthesis of electroactive nanomaterials by using plant-derived natural products. J. Electroanal. Chem..

[15] Jianrong C., Yuqing M., Nongyue H., Xiaohua W., Sijiao L. (2004). Nanotechnology and biosensors. Biotechnol. Adv..

[16] Oprea D., Sanz C.G., Barsan M.M., Enache T.A. (2022). PC-12 Cell Line as a Neuronal Cell Model for Biosensing Applications. Biosensors.

[17] El-Aassar M.R., Ibrahim O.M., Fouda M.M.G., El-Beheri N.G., Agwa M.M. (2020). Wound healing of nanofiber comprising Polygalacturonic/Hyaluronic acid embedded silver nanoparticles: In-vitro and in-vivo studies. Carbohydr. Polym..

[18] Hetta H.F., Al-Kadmy I.M.S., Khazaal S.S., Abbas S., Suhail A., El-Mokhtar M.A., Abd Ellah N.H., Ahmed E.A., Abd-ellatief R.B., El-Masry E.A. (2021). Antibiofilm and antivirulence potential of silver nanoparticles against multidrug-resistant *Acinetobacter baumannii*. Sci. Rep..

[19] Sreelekha E., Bini G., Aswathi S., Sajina N., Beena M. (2021). A comparative study on the synthesis, characterization, and antioxidant activity of green and chemically synthesized silver nanoparticles. BioNanoScience.

[20] Scandurra A., Censabella M., Gulino A., Grimaldi M.G., Ruffino F. (2022). Gold nanoelectrode arrays dewetted onto graphene paper for selective and direct electrochemical determination of glyphosate in drinking water. Sens. Bio-Sens. Res..

[21] Sharma V.K., Yngard R.A., Lin Y. (2009). Silver nanoparticles: Green synthesis and their antimicrobial activities. Adv. Colloid Interface Sci..

[22] Maruccio G., Narang J. (2022). Electrochemical Sensors: From Working Electrodes to Functionalization and Miniaturized Devices.

[23] Herrera-Domínguez M., Morales-Luna G., Mahlknecht J., Cheng Q., Aguilar-Hernández I., Ornelas-Soto N. (2023). Optical Biosensors and Their Applications for the Detection of Water Pollutants. Biosensors.

[24] Emami T., Madani R., Golchinfar F., Shoushtary A., Amini S.M. (2015). Comparison of Gold Nanoparticle Conjugated Secondary Antibody with Non-Gold Secondary Antibody in an ELISA Kit Model. Monoclon. Antibodies Immunodiagn. Immunother..

[25] Jamkhande P.G., Ghule N.W., Bamer A.H., Kalaskar M.G. (2019). Metal nanoparticles synthesis: An overview on methods of preparation, advantages and disadvantages, and applications. J. Drug Deliv. Sci. Technol..

[26] Thakkar K.N., Mhatre S.S., Parikh R.Y. (2010). Biological synthesis of metallic nanoparticles. Nanomed. Nanotechnol. Biol. Med..

[27] Jasim N.O., Hasan T.K., Flieh H. (2018). Characterization of silver nano particles synthesized by leaves green tea extract. J. Glob. Pharma Technol..

[28] Malik S., Niazi M., Khan M., Rauff B., Anwar S., Amin F., Hanif R. (2023). Cytotoxicity study of gold nanoparticle synthesis using *Aloe vera*, honey, and *Gymnema sylvestre* leaf extract. ACS Omega.

[29] Lee J., Park E.Y., Lee J. (2014). Non-toxic nanoparticles from phytochemicals: Preparation and biomedical application. Bioprocess Biosyst. Eng..

[30] Park Y., Hong Y.N., Weyers A., Kim Y.S., Linhardt R.J. (2011). Polysaccharides and phytochemicals: A natural reservoir for the green synthesis of gold and silver nanoparticles. IET Nanobiotechnol..

[31] Khan M.A., Khan T., Nadhman A. (2016). Applications of plant terpenoids in the synthesis of colloidal silver nanoparticles. Adv. Colloid Interface Sci..

[32] Venkata A.L.K., Sivaram S., Sajeet M., Sanjay P.M., Srilakshman G., Muthuraman M.S. (2022). Review on terpenoid mediated nanoparticles: Significance, mechanism, and biomedical applications. Adv. Nat. Sci. Nanosci. Nanotechnol..

[33] Keijok W.J., Pereira R.H.A., Alvarez L.A.C., Prado A.R., da Silva A.R., Ribeiro J., de Oliveira J.P., Guimarães M.C.C. (2019). Controlled biosynthesis of gold nanoparticles with *Coffea arabica* using factorial design. Sci. Rep..

[34] Baghaienezhad M., Boroghani M., Anabestani R. (2020). Silver nanoparticles synthesis by coffee residues extract and their antibacterial activity. Nanomed. Res. J..

[35] Zameer S., Yamamoto Y. (2011). Carbohydrate-directed synthesis of silver and gold nanoparticles: Effect of the structure of carbohydrates and reducing agents on the size and morphology of the composites. Carbohydr. Res..

[36] Maruyama T., Fujimoto Y., Maekawa T. (2015). Synthesis of gold nanoparticles using various amino acids. J. Colloid Interface Sci..

[37] Rashid M.U., Khairul H.B., Quayum M.E. (2013). Synthesis of silver nano particles (Ag-NPs) and their uses for quantitative analysis of vitamin C tablets. Dhaka Univ. J. Pharm. Sci..

[38] Daizy P. (2010). Honey mediated green synthesis of silver nanoparticles. Spectrochim. Acta Part A Mol. Biomol. Spectrosc..

[39] Willner I., Baron R., Willner B. (2006). Growing metal nanoparticles by enzymes. Adv. Mater..

[40] Khan A.U., Wei Y., Ahmad A., Khan Z.U.H., Tahir K., Khan S.U., Muhammad N., Khan F.U., Yuan Q. (2016). Enzymatic browning reduction in white cabbage, potent antibacterial and antioxidant activities of biogenic silver nanoparticles. J. Mol. Liq..

[41] Tarannum N., Divya and Gautam Y.K. (2019). Facile green synthesis and applications of silver nanoparticles: A state-of-the-art review. RSC Adv..

[42] Lee S.H., Jun B.-H. (2019). Silver nanoparticles: Synthesis and application for nanomedicine. Int. J. Mol. Sci..

[43] Chen H., Kou X., Yang Z., Ni W., Wang J. (2008). Shape-and size-dependent refractive index sensitivity of gold nanoparticles. Langmuir.

[44] Yang Z.-X., Zhong W., Au C., Wanga J.-Y., Du Y.-W. (2011). An environment-benign solvothermal method for the synthesis of flower-like hierarchical nickel and zinc compounds and their transformation to nanoporous NiO and ZnO. CrystEngComm.

[45] Mousavi S.M., Behbudi G., Gholami A., Hashemi S.A., Nejad Z.M., Bahrani S., Chiang W.-H., Wei L.C., Omidifar N. (2022). Shape-controlled synthesis of zinc nanostructures mediating macromolecules for biomedical applications. Biomater. Res..

[46] Anu R., Yadav K., Jagadevan S. (2020). A comprehensive review on green synthesis of nature-inspired metal nanoparticles: Mechanism, application and toxicity. J. Clean. Prod..

[47] Samson R., Navale G.R., Dharne M.S. (2020). Biosensors: Frontiers in rapid detection of COVID-19. 3 Biotech.

[48] Wang S., Payne G.F., Bentley W.E. (2020). Quorum sensing communication: Molecularly connecting cells, their neighbors, and even devices. Annu. Rev. Chem. Biomol. Eng..

[49] Rohiwal S.S., Dvorakova N., Klima J., Vaskovicova M., Senigl F., Slouf M., Pavlova E., Stepanek P., Babuka D., Benes H. (2020). Polyethylenimine based magnetic nanoparticles mediated non-viral CRISPR/Cas9 system for genome editing. Sci. Rep..

[50] Manhas J., Edelstein H.I., Leonard J.N., Morsut L. (2022). The evolution of synthetic receptor systems. Nat. Chem. Biol..

[51] McCarty N.S., Graham A.E., Studená L., Ledesma-Amaro R. (2020). Multiplexed CRISPR technologies for gene editing and transcriptional regulation. Nat. Commun..

[52] Mousavi P.S., Smith S.J., Chen J.B., Karlikow M., Tinafar A., Robinson C., Liu W., Ma D., Green A.A., Kelley S.O. (2020). A multiplexed, electrochemical interface for gene-circuit-based sensors. Nat. Chem..

[53] Ahmar S., Mahmood T., Fiaz S., Mora-Poblete F., Shafique M.S., Chattha M.S., Jung K.-H. (2021). Advantage of nanotechnology-based genome editing system and its application in crop improvement. Front. Plant Sci..

[54] Sun J., Lu Y., He L., Pang J., Yang F., Liu Y. (2019). Colorimetric sensor array based on gold nanoparticles: Design principles and recent advances. Trends Anal. Chem..

[55] Montes-Garcia V., Squillaci M.A., Diez-Castellnou M., Ong Q.K., Stellacci F., Samori P. (2021). Chemical sensing with Au and Ag nanoparticles. Chem. Soc. Rev..

[56] Al-Zahrani S., Astudillo-Calderón S., Pintos B., Pérez-Urria E., Manzanera J.A., Martín L., Gomez-Garay A. (2021). Role of Synthetic Plant Extracts on the Production of Silver-Derived Nanoparticles. Plants.

[57] Beck F., Loessl M., Baeumner A.J. (2023). Signaling strategies of silver nanoparticles in optical and electrochemical biosensors: Considering their potential for the point-of-care. Microchim. Acta.

[58] Prosposito P., Burratti L., Venditti I. (2020). Silver Nanoparticles as Colorimetric Sensors for Water Pollutants. Chemosensors.

[59] Forough M., Farhadi K. (2010). Biological and green synthesis of silver nanoparticles. Turk. J. Eng. Environ. Sci..

[60] Farhadi K., Forough M., Molaei R., Hajizadeh S., Rafipour A. (2012). Highly selective Hg2+ colorimetric sensor using green synthesized and unmodified silver nanoparticles. Sens. Actuators B Chem..

[61] Sebastian M., Aravind A., Mathew B. (2018). Green Silver Nanoparticles Based Dual Sensor for Toxic Hg(II) Ions. Nanotechnology.

[62] Kadam J., Dhawal P., Barve S., Kakodkar S. (2020). Green synthesis of silver nanoparticles using cauliflower waste and their multifaceted applications in photocatalytic degradation of methylene blue dye and Hg^2+^ biosensing. SN Appl. Sci..

[63] Mavaei M., Chahardoli A., Fattahi A., Khoshroo A. (2021). A Simple Method for Developing a Hand-Drawn Paper-Based Sensor for Mercury; Using Green Synthesized Silver Nanoparticles and Smartphone as a Hand-Held-Device for Colorimetric Assay. Glob. Chall..

[64] Ramachandiran D., Rajesh K. (2022). Selective colorimetric and fluorimertic sensor of Hg(II) ion from silver nanoparticles using *Acacia chundra* leaves extract. Mater. Chem. Phys..

[65] Bhagat S., Shaikh H., Nafady A., Sirajuddin, Sherazi S.T.H., Bhanger M.I., Shah M.R., Abro M.I., Memon R., Bhagat R. (2022). Trace Level Colorimetric Hg^2+^ Sensor Driven by Citrus japonica Leaf Extract Derived Silver Nanoparticles: Green Synthesis and Application. J. Clust. Sci..

[66] Punnoose M.S., Bijimol D., Abraham T., Plathanam N.J., Mathew B. (2021). Green Synthesized Unmodified Silver Nanoparticles as Reproducible Dual Sensor for Mercuric Ions and Catalyst to Abate Environmental Pollutants. BioNanoScience.

[67] Gangarapu M., Anaikutti P., Sarangapany S. (2020). Sustainable Utilization of Molasses Towards Green Synthesis of Silver Nanoparticles for Colorimetric Heavy Metal Sensing and Catalytic Applications. J. Clust. Sci..

[68] Edison T.N.J.I., Atchudan R., Lee Y.R. (2016). Optical Sensor for Dissolved Ammonia Through the Green Synthesis of Silver Nanoparticles by Fruit Extract of *Terminalia chebula*. J. Clust. Sci..

[69] Pandey S., Goswami G.K., Nanda K.K. (2012). Green synthesis of biopolymer–silver nanoparticle nanocomposite: An optical sensor for ammonia detection. Int. J. Biol. Macromol..

[70] Srikhao N., Kasemsiri P., Lorwanishpaisarn N., Okhawilai M. (2021). Green synthesis of silver nanoparticles using sugarcane leaves extract for colorimetric detection of ammonia and hydrogen peroxide. Res. Chem. Intermed..

[71] Aadila K.R., Pandey N., Mussatto S.I., Jha H. (2019). Green synthesis of silver nanoparticles using acacia lignin, their cytotoxicity, catalytic, metal ion sensing capability and antibacterial activity. J. Environ. Chem. Eng..

[72] Bindhu M.R., Umadevi M., Esmail G.A., Al-Dhabi N.A., Arasu M.V. (2020). Green synthesis and characterization of silver nanoparticles from Moringa oleifera flower and assessment of antimicrobial and sensing properties. J. Photochem. Photobiol. B Biol..

[73] Aravind A., Sebastian M., Mathew B. (2018). Green silver nanoparticles as a multifunctional sensor for toxic Cd(II) ions. New J. Chem..

[74] Aravind A., Sebastian M., Mathew B. (2018). Green synthesized unmodified silver nanoparticles as a multi-sensor for Cr(III) ions. Environ. Sci. Water Res. Technol..

[75] Tagad C.K., Dugasani S.R., Aiyer R., Park S., Kulkarni A., Sabharwal S. (2013). Green synthesis of silver nanoparticles and their application for the development of optical fiber based hydrogen peroxide sensor. Sens. Actuators B.

[76] Elgamouz A., Idriss H., Nassab C., Bihi A., Bajou K., Hasan K., Haija M.A., Patole S.P. (2020). Green Synthesis, Characterization, Antimicrobial, Anti-Cancer, and Optimization of Colorimetric Sensing of Hydrogen Peroxide of Algae Extract Capped Silver Nanoparticles. Nanomaterials.

[77] Chandraker S.K., Lal M., Kumar A., Shukla R. (2022). *Justicia adhatoda* L. mediated green synthesis of silver nanoparticles and assessment of their antioxidant, hydrogen peroxide sensing and optical properties. Mater. Technol..

[78] Mahadevan S., Vijayakumar S., Arulmozhi P. (2017). Green synthesis of silver nano particles from *Atalantia monophylla* (L.) Correa leaf extract, their antimicrobial activity and sensing capability of H_2_O_2_. Microb. Pathog..

[79] Alipilakkotte S., Sreejith L. (2018). Green synthesized PLA/silver nanoparticle probe for sensing of hydrogen peroxide in biological samples. Mater. Lett..

[80] Salazar P., Fernández I., Rodríguez M.C., Creus A.H., González-Mora J.L. (2019). One-step green synthesis of silver nanoparticle-modified reduced graphene oxide nanocomposite for H_2_O_2_ sensing applications. J. Electroanal. Chem..

[81] Nam N.T.H., Dat N.M., Hai N.D., Huong L.M., Tai L.T., Dat N.T., An H., Hung P.N.P., Truong N.T., Son N.T. (2023). Green synthesis of silver@graphene oxide nanocomposite for antibacterial, cytotoxicity assessment, and hydrogen peroxide electro-sensing. New J. Chem..

[82] Turunc E., Kahraman O., Binzet R. (2021). Green synthesis of silver nanoparticles using pollen extract: Characterization, assessment of their electrochemical and antioxidant activities. Anal. Biochem..

[83] Zamarchi F., Vieira I.C. (2021). Determination of paracetamol using a sensor based on green synthesis of silver nanoparticles in plant extract. J. Pharm. Biomed. Anal..

[84] Verma D., Chauhan D., Mukherjee M.D., Ranjan K.R., Yadav A.K., Solanki P.R. (2021). Development of MWCNT decorated with green synthesized AgNps-based electrochemical sensor for highly sensitive detection of BPA. J. Appl. Electrochem..

[85] Verma D., Dhiman T.K., Mukherjee M.D., Solanki P.R. (2021). Electrophoretically Deposited Green Synthesized Silver Nanoparticles Anchored in Reduced Graphene Oxide Composite Based Electrochemical Sensor for Detection of Bisphenol A. J. Electrochem. Soc..

[86] Jaballah M.B., Messaoud N.B., Dridi C. (2022). Development of cost-effective and sustainable sensing nanoplatform based on green AgNPs for the determination of BPA in water. J. Mater. Sci. Mater. Electron..

[87] Teimuri-Mofrad R., Hadi R., Tahmasebi B., Farhoudian S., Mehravar M., Nasiri R. (2017). Green synthesis of gold nanoparticles using plant extract: Mini-Review. Nanochem. Res..

[88] Lee K.X., Shameli K., Yew Y.P., Teow S.Y., Jahangirian H., Rafiee-Moghaddam R., Webster T.J. (2020). Recent Developments in the Facile Bio-Synthesis of Gold Nanoparticles (AuNPs) and Their Biomedical Applications. Int. J. Nanomed..

[89] Pandey S., Goswami G.K., Nanda K.K. (2013). Green synthesis of polysaccharide/gold nanoparticle nanocomposite: An efficient ammonia sensor. Carbohydr. Polym..

[90] Gangapuram B.R., Bandi R., Dadigala R., Kotu G.M., Guttena V. (2017). Facile Green Synthesis of Gold Nanoparticles with Carboxymethyl Gum Karaya, Selective and Sensitive Colorimetric Detection of Copper(II) Ions. J. Clust. Sci..

[91] Bahram M., Mohammadzadeh E. (2014). Green synthesis of gold nanoparticles with willow tree bark extract: A sensitive colourimetric sensor for cysteine detection. Anal. Methods.

[92] Ashikbayeva Z., Bekmurzayeva A., Myrkhiyeva Z., Assylbekova N., Atabaev T.S., Tosi D. (2023). Green-synthesized gold nanoparticle-based optical fiber ball resonator biosensor for cancer biomarker detection. Opt. Laser Technol..

[93] Li H., Hu W., Hassan M.M., Zhang Z., Chen Q. (2019). A facile and sensitive SERS-based biosensor for colormetric detection of acetamiprid in green tea based on unmodified gold nanoparticles. J. Food Meas. Charact..

[94] Nazarpour S., Hajian R., Sabzvaria M.H. (2020). A novel nanocomposite electrochemical sensor based on green synthesis of reduced graphene oxide/gold nanoparticles modified screen printed electrode for determination of tryptophan using response surface methodology approach. Microchem. J..

[95] Tabrizia M.A., Varkanib J.N. (2014). Green synthesis of reduced graphene oxide decorated with gold nanoparticles and its glucose sensing application. Sens. Actuators B Chem..

[96] Karthik R., Govindasamy M., Chen S.-M., Mani V., Lou B.-S., Devasenathipathy R., Hou Y.-S., Elangovan A. (2016). Green synthesized gold nanoparticles decorated graphene oxide for sensitive determination of chloramphenicol in milk, powdered milk, honey and eye drops. J. Colloid Interface Sci..

[97] González-Fuentes F.J., Molina G.A., Silva R., López-Miranda J.L., Esparza R., Hernandez-Martinez A.R., Estevez M. (2020). Developing a CNT-SPE Sensing Platform Based on Green Synthesized AuNPs, Using *Sargassum* sp.. Sensors.

[98] Messai Y., Bezzi H., Hellal N., Belbacha W., Messali S., Belghidoum A., Foudia M., Schmutz M., Blanck C., Derafa W. (2022). A novel green synthesized NiO nanoparticles modified glassy carbon electrode for non-enzymatic glucose sensing. Microchem. J..

[99] Mahdavi B., Paydarfard S., Rezaei-Seresht E., Baghayeri M., Nodehi M. (2021). Green synthesis of NiONPs using *Trigonella subenervis* extract and its applications as a highly efficient electrochemical sensor, catalyst, and antibacterial agent. Appl. Organomet. Chem..

[100] Wang L., Zhang Y., Yu J., He J., Yang H., Ye Y., Song Y. (2017). A green and simple strategy to prepare graphene foam-like three-dimensional porous carbon/Ni nanoparticles for glucose sensing. Sens. Actuators B Chem..

[101] Likasari I.D., Astuti R.W., Yahya A., Isnaini N., Purwiandono G., Hidayat H., Wicaksono W.P., Fatimah I. (2021). NiO nanoparticles synthesized by using *Tagetes erecta* L leaf extract and their activities for photocatalysis, electrochemical sensing, and antibacterial features. Chem. Phys. Lett..

[102] Rajith Kumar C.R., Betageri V.S., Nagaraju G., Pujar G.H., Suma B.P., Latha M.S. (2020). Photocatalytic, nitrite sensing and antibacterial studies of facile bio-synthesized nickel oxide nanoparticles. J. Sci. Adv. Mater. Devices.

[103] Das T.R., Sharma P.K. (2020). Hydrothermal-assisted green synthesis of Ni/Ag@rGO nanocomposite using Punica granatum juice and electrochemical detection of ascorbic acid. Microchem. J..

[104] Kulkarni S.S., Shirsat M.D. (2015). Optical and structural properties of zinc oxide nanoparticles. Int. J. Adv. Res. Phys. Sci..

[105] Morozov I.G., Belousova O.V., Ortega D., Mafina M.K., Kuznetcov M.V. (2015). Structural, optical, XPS and magnetic properties of Zn particles capped by ZnO nanoparticles. J. Alloys Compd..

[106] Darvishi E., Kahrizi D., Arkan E. (2019). Comparison of different properties of zinc oxide nanoparticles synthesized by the green (using *Juglans regia* L. leaf extract) and chemical methods. J. Mol. Liq..

[107] Muthuchamy N., Atchudan R., Edison T.N.J.I., Perumal S., Lee Y.R. (2018). High-performance glucose biosensor based on green synthesized zinc oxide nanoparticle embedded nitrogen-doped carbon sheet. J. Electroanal. Chem..

[108] Sharma D., Sabela M.I., Kanchi S., Bisetty K., Skelton A.A., Honarparvar B. (2018). Green synthesis, characterization and electrochemical sensing of silymarin by ZnO nanoparticles: Experimental and DFT studies. J. Electroanal. Chem..

[109] Wicaksono W.P., Fadilla N.I., Zamar A.A., Fadillah G., Anugrahwati M., Anas A.K., Kadja G.T.M. (2022). Formaldehyde electrochemical sensor using graphite paste-modified green synthesized zinc oxide nanoparticles. Inorg. Chem. Commun..

[110] Joshi L.P., Khatri B.V., Gyawali S., Gajurel S., Chaudhary D.K. (2021). Green Synthesis of Zinc Oxide Nanoparticles Using Ixora Coccinea Leaf Extract for Ethanol Vapour Sensing. J. Phys. Sci..

[111] Narayana A., Bhat S.A., Fathima A., Lokesh S.V., Suryad S.G., Yelamaggad C.V. (2020). Green and low-cost synthesis of zinc oxide nanoparticles and their application in transistor-based carbon monoxide sensing. RSC Adv..

[112] Saleh T.A., Fadillah G. (2023). Green synthesis protocols, toxicity, and recent progress in nanomaterial-based for environmental chemical sensors applications. Trends Environ. Anal. Chem..

[113] Zagal-Padilla C.K., Diaz-Gómez C., Gamboa S.A. (2022). Electrochemical characterization of a plasmonic effect ethanol sensor based on two-dimensional ZnO synthesized by green chemistry. Mater. Sci. Semicond. Process..

[114] Rohatgi V., Challagulla N.V., Pudake R.N. (2021). Volatile Organic Compounds (VOCs) Sensors for Stress Management in Crops. Biosensors in Agriculture: Recent Trends and Future Perspectives.

[115] Gharari Z., Hanachi P., Walker T.R. (2022). Green synthesized Ag-nanoparticles using Scutellaria multicaulis stem extract and their selective cytotoxicity against breast cancer. Anal. Biochem..

